# Prognosis and Treatment Approach for Stage 1 Retinal Angiomatous Proliferation Lesions: A Case Series With Long-Term Follow-Up

**DOI:** 10.7759/cureus.107009

**Published:** 2026-04-14

**Authors:** Dimitris Karagiannis, Nikolaos Bouratzis, Loukas Kontomichos, Georgios Batsos, Efstratios Paroikakis

**Affiliations:** 1 Second Ophthalmology Department, Specialized Eye Hospital, Ophthalmiatreio Athinon, Athens, GRC; 2 Medical Retina Department, Moorfields Eye Hospital, London, GBR

**Keywords:** anti-vegf treatment, oct (optical coherence tomography), retinal angiomatous proliferation (rap), type 3 choroidal neovascularization (cnv), wet amd (age-related macular degeneration)

## Abstract

Retinal angiomatous proliferation (RAP) accounts for approximately 10%-20% of all neovascular (wet) age-related macular degeneration and is classically classified into three stages, with stage 1 representing the earliest form of disease. Despite its clinical relevance, limited evidence exists on the long-term prognosis of treatment-naïve stage 1 RAP, particularly regarding recurrence patterns and the development of macular atrophy or fibrosis over extended follow-up. This retrospective case series evaluates outcomes in four patients diagnosed with stage 1 RAP using optical coherence tomography (OCT) and fluorescein angiography. All patients were planned to receive a loading regimen of three monthly intravitreal aflibercept injections, followed by monthly review for at least 24 months. The loading phase was modified according to findings at each visit. At every appointment, best-corrected visual acuity (BCVA), slit-lamp examination, and OCT assessment of macular anatomy were performed, with recurrence defined as clinical and/or OCT evidence of exudative activity warranting re-treatment. Over the follow-up period, all four patients demonstrated stable or improved BCVA after anti-vascular endothelial growth factor (VEGF) therapy. Two patients had no recurrences across 24 months, while two patients each experienced a single recurrence, occurring at seven months and 24 months, respectively. Importantly, no eyes developed macular atrophy or fibrotic scarring during the observation period, and visual acuity remained stable or improved in all cases. These findings support the concept that early identification and prompt treatment of stage 1 RAP may be associated with a low recurrence burden and a favourable anatomic course, potentially limiting progression to atrophy or fibrosis and preserving long-term vision. They also suggest that a personalised management strategy, combining an initial loading phase with close surveillance and re-treatment only upon recurrence, may be effective in carefully selected early-stage RAP patients and could reduce overall injection burden. Larger studies with longer follow-up are required to confirm these preliminary observations and to better define stage-specific treatment strategies for RAP.

## Introduction

Neovascular age-related macular degeneration (nAMD) is a devastating sight-threatening disease and the most common cause of blindness in the developed world, characterised by pathological choroidal neovascularisation (CNV), which is classified into three subtypes based on the anatomical location of the neovascular complex. Retinal angiomatous proliferation (RAP), also referred to as type 3 choroidal neovascularisation (CNV type 3), represents 10%-20% of all neovascular AMD cases and is a distinct subtype characterised by intraretinal neovascularisation originating from the deep retinal capillary plexus, which extends posteriorly into the subretinal space and may ultimately anastomose with choroidal neovascularisation to form a retinal-choroidal anastomosis. This pathological process is typically associated with significant intraretinal and subretinal fluid, pigment epithelial detachment, and progressive photoreceptor loss, and carries a poorer visual prognosis and higher risk of bilateral involvement compared to classic CNV subtypes [1].

Yannuzzi et al. classified RAP lesions into three stages using fluorescein angiography (FA) and indocyanine green angiography (ICGA), based on the anatomical origin and extent of the neovascular process [2]. Stage 1 is defined by intraretinal neovascularisation alone. Stage 2 is characterised by retinal-retinal anastomoses and intraretinal oedema and may be associated with a serous pigment epithelial detachment (PED) and/or subretinal neovascularisation. Stage 3 is marked by the presence of retinochoroidal anastomoses and true choroidal neovascularisation [2].

A separate optical coherence tomography (OCT)-based classification was subsequently proposed by Su et al. [3]. Stage 1 comprises a large intraretinal hyperreflective lesion associated with cystoid macular oedema, without disruption of the outer retinal layers. Stage 2 is defined by outer retinal disruption, which occurs in conjunction with retinal pigment epithelium (RPE) disruption in the majority of cases. Stage 3 describes an intraretinal hyperreflective lesion that extends through the RPE to vascularise a drusenoid PED, generating a serous component within the pigment epithelial detachment [3].

Although numerous studies have examined the prognosis of type 3 CNV, outcomes appear to vary considerably across disease stages. Notably, studies specifically including patients with stage 1 RAP lesions and reporting long-term follow-up data remain scarce. The primary objective of this study is therefore to highlight the importance of early detection of RAP lesions, supported by the long-term follow-up outcomes of four patients presenting with early-stage disease.

## Case presentation

We report four cases (four eyes) with treatment-naïve type 3 choroidal neovascularisation (RAP) stage 1, diagnosed after full ophthalmological examination, including optical coherence tomography and fluorescein angiography or OCT angiography (OCTA). An intraretinal hyperreflective lesion along with intraretinal fluid was noticed in all cases without any disruption of the outer retinal layers. Best-corrected visual acuity (BCVA) using the Snellen Charts was measured at baseline and at each monthly follow-up visit. Using the classification system proposed by Su et al., utilising the OCT, we characterised all lesions as stage 1 [3]. All patients were initially scheduled to receive a loading regimen of three monthly intravitreal aflibercept injections, followed by monthly review for at least 24 months. However, the loading phase was adjusted on an individual basis according to the clinical findings at each visit. Monthly follow-up included BCVA, slit-lamp examination, and optical coherence tomography angiography. Treatment response and retreatment decisions were guided by fundus and OCT findings and changes in visual acuity. The presence or recurrence of fluid, exudation, or intraretinal haemorrhage, as well as a decline in visual acuity, were the primary criteria for treatment. A good prognosis was defined as the presence of all of the following: preservation or improvement of BCVA, minimal or no recurrences, and no development of extensive macular atrophy and/or fibrosis. Patients' other eyes did not exhibit any signs of choroidal neovascularisation during the follow-up period (Table 1). Written consent was obtained from all patients.

**Table 1:** Patients characteristics REC: recurrence; 1st REC: months after the last injection of the loading dose, and loading dose was different for each patient; BCVA: Best-corrected visual acuity.

	Age	M/F	Eye	Injections Until Dry	1st REC (Month)	Number of REC	BCVA (Baseline)	BCVA (24 Months)	Follow-up Period
Patient 1	84	F	L	2	0	0	6/15	6/9.5	24
Patient 2	89	F	R	3	0	0	6/30	6/18	24
Patient 3	89	M	R	1	24	1	6/24	6/18	28
Patient 4	85	M	R	3	4	1	6/12	6/12	26

Case 1

**Figure 1:**
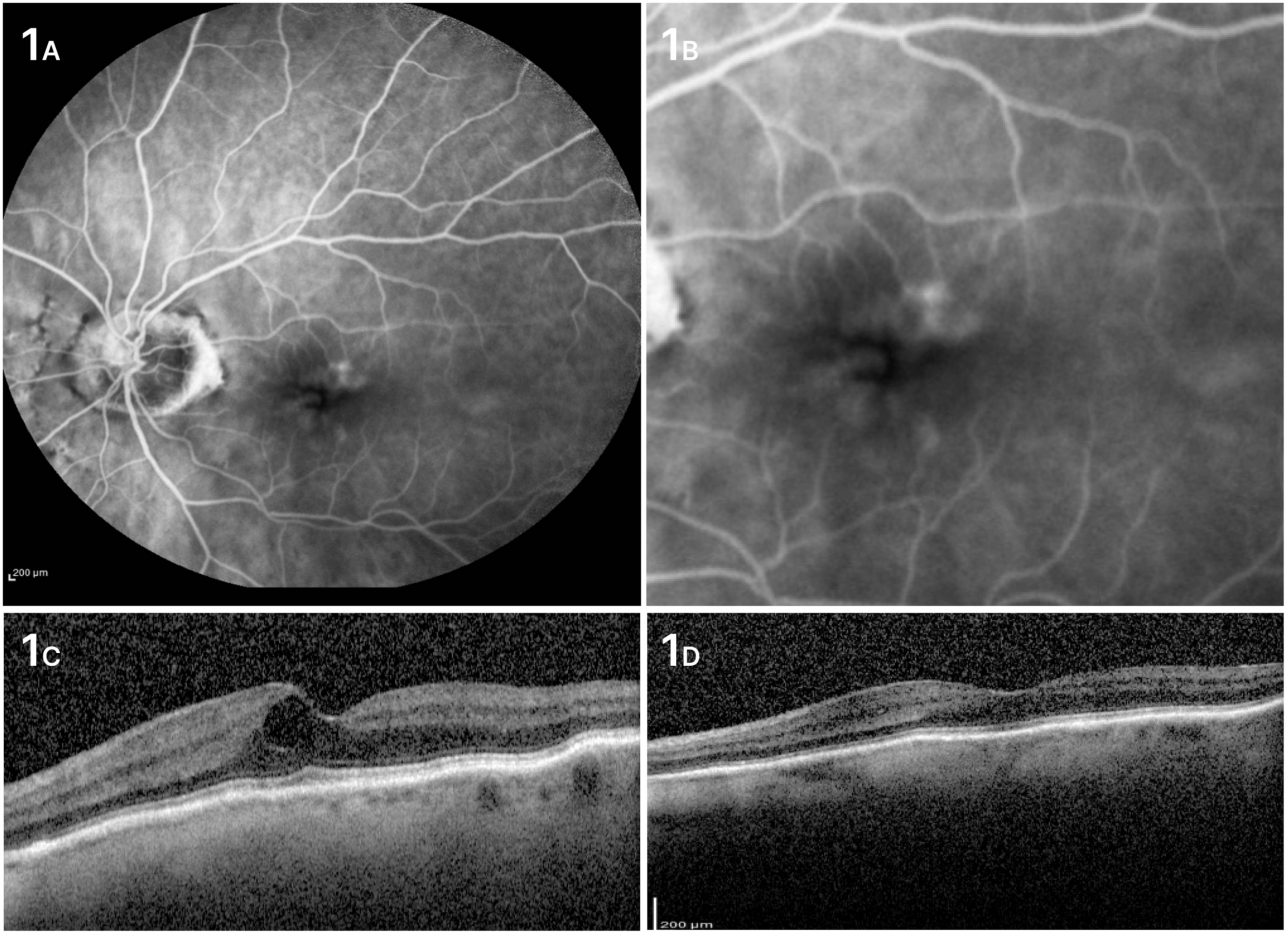
Case 1 A. Focal area of intense intraretinal hyperfluorescence (hot spot) in fluorescein angiography early phase. B. Magnification in the hyperfluorescent area where the retinal-retinal anastomoses can be observed. C. OCT of the left eye at initial diagnosis with an intraretinal cyst. D. OCT of the left eye at a 24-month follow-up period. OCT: optical coherence tomography.

An 84-year-old female presented with painless loss of vision in her left eye. Best-corrected visual acuity at initial visit was 6/9.5 in her right eye and 6/15 in her left eye. Patient was pseudophakic and was suffering from early glaucoma. Intraocular pressure (IOP) was controlled efficiently only with eyedrops (prostaglandin analogues). After thorough examination using optical coherence tomography and fluorescein angiography, the diagnosis of retinal angiomatous proliferation was made in patient's left eye. The RAP lesion was characterised as stage 1 based on the classification proposed by Su et al. using the OCT (Figures 1A-1D) [3]. Two monthly anti-vascular endothelial growth factor (VEGF) injections were needed until the macula was dry. We decided not to administer the third injection as a loading dose and monitor the patient closely. BCVA was improved significantly (6/9.5) after two injections, and no recurrence was noted during the follow-up period (24 months).

Case 2

**Figure 2:**
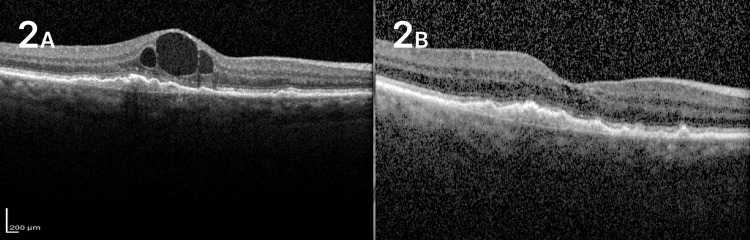
Case 2 A. Initial OCT of the right eye shows intraretinal cysts and a pre-existing drusenoid retinal pigment epithelial elevation (PED). B. OCT of the same eye after a 24-month follow-up that demonstrates no intraretinal fluid, no fibrosis, and no macular atrophy. OCT: optical coherence tomography.

An 89-year-old female was referred to our medical retina department after she was diagnosed with wet AMD in her right eye. Patient was pseudophakic and had no past ocular history. BCVA at presentation was 6/30 in her right eye and 6/12 in her left eye. OCT of the right eye illustrated an intraretinal cyst with an underlying drusenoid retinal pigment epithelium elevation (Figure 2A). Since there was no serous or fibrovascular (PED), the lesion was classified as an advanced stage 1 RAP lesion. Dry macula was achieved after three monthly intravitreal injections with aflibercept. The patient demonstrated only one recurrence after 24 months of follow-up without any severe complications in her visual acuity. Visual acuity before recurrence was 6/18 and remained stable after treatment. No macular atrophy and/or fibrosis was noted after 28 months of follow-up (Figure 2B).

Case 3

**Figure 3:**
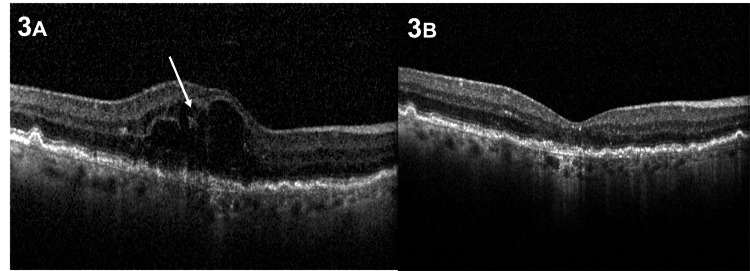
Case 3 A. OCT at initial visit shows extensive intraretinal fluid with underlying drusen and no adjacent pigment epithelial detachment. The RAP lesion is depicted as a hyperreflective lesion in the inner retina (white arrow). B. OCT of the right eye at a 28-month follow-up visit with underlying drusenoid abnormalities of the RPE. OCT: optical coherence tomography; RAP: retinal angiomatous proliferation; RPE: retinal pigment epithelium.

An 89-year-old male was diagnosed with a stage 1 RAP lesion in his right eye. The OCT scan depicted a hyperreflective intraretinal lesion without disruption of the outer retinal layers (Figure 3A). At presentation, best-corrected visual acuity (BCVA) was 6/24 in the right eye and 6/12 in the left eye. Following a single intravitreal injection, there was complete resolution of intraretinal fluid. The patient declined further treatment and opted for close monthly monitoring. BCVA improved to 6/18 after the injection, and no recurrence was observed over a 28-month follow-up period. (Figure 3B). No fibrosis or extensive macular atrophy was noted on the scans of the last follow-up visit.

Case 4

**Figure 4:**
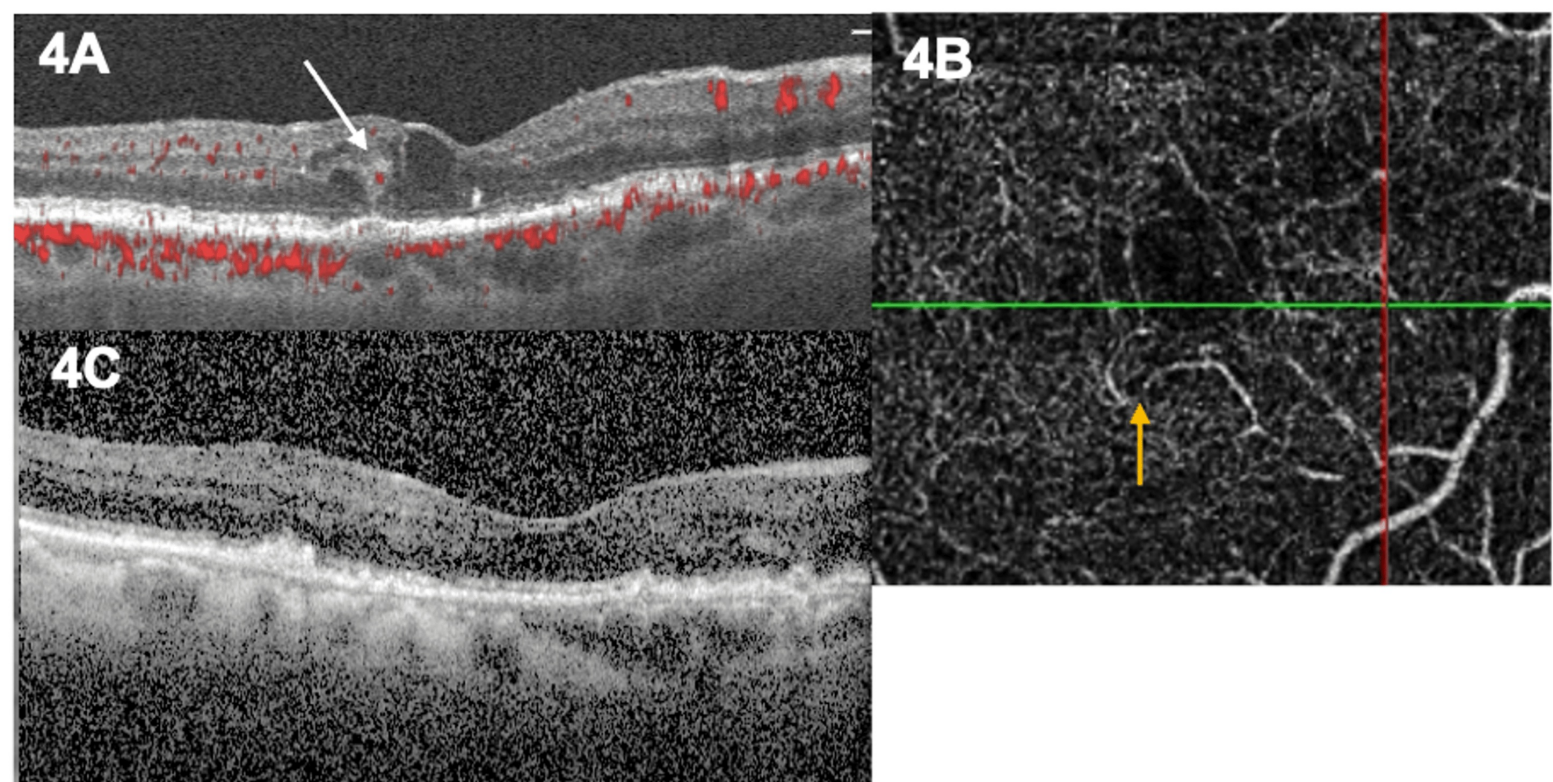
Case 4 A. OCT B-scan of the right eye shows intraretinal fluid and a hyperreflective lesion in the deep capillary plexus. Blood flow is depicted in the lesion (white arrow). B. OCTA of the same eye, deep capillary plexus: retinal-retinal anastomoses are depicted (orange arrow). C. OCT of the right eye at a 26-month follow-up. No fluid is present while no signs of atrophy are observed. OCT: optical coherence tomography; OCTA: OCT angiography.

An 85-year-old male was treated with a loading course of intravitreal aflibercept injections following the diagnosis of a stage 1 RAP lesion in his right eye. The diagnosis was established using both OCT and OCT angiography (Figure 4). The OCT B-scan demonstrated an intraretinal hyperreflective lesion with flow (Figure 4A), while OCTA revealed retinal-retinal anastomoses within the deep retinal capillary plexus, without involvement of the outer retina or choroid (Figure 4B). At presentation, BCVA was 6/12 in the right eye and 6/6 in the left eye. BCVA remained stable at 6/12 following three consecutive injections, with no evidence of intraretinal or subretinal fluid on OCT. However, four months after the third injection (seven months from initial presentation), intraretinal fluid recurred, prompting a fourth injection. No further recurrences were observed during a 26-month follow-up period, and BCVA remained stable (Figure 4C).

## Discussion

Time and number of recurrences in neovascular age-related macular degeneration (nAMD) are major prognostic factors in the final outcome of the disease. Two retrospective studies published by Kim et al. report that the mean time of the first recurrence after a loading dose of three consecutive anti-VEGF injections is 6.6 and 5.3 months, respectively [4-5]. However, both studies included only patients with advanced stages (stages 2 and 3) RAP lesions. Currently, there are no published data on the first recurrence time in patients exhibiting stage 1 RAP lesions. Kim et al. also suggest that patients with an early recurrence (<6 months) tend to develop multiple recurrences, while a second recurrence was not noted in 64.3% of the patients who experienced the first recurrence at least six months after the third anti-VEGF injection [5]. The follow-up period of the patients in these studies was relatively short (12 months); thus, estimating the exact recurrence rate in those patients is rather difficult. Three of our patients exhibited no recurrence in the first year, while one of them developed fluid after 24 months. One patient developed a recurrence seven months after the initial visit and reported no other recurrence for the rest of the follow-up period (24 months).

Retinal angiomatous proliferation is more aggressive than types 1 and 2 CNV with more rapid progression and worse visual outcomes at late stages of the disease. Long-term visual outcomes in patients with RAP depend mostly on the development of macular atrophy, subretinal haemorrhage, and fibrotic scars [6]. Numerous studies suggest that the risk of developing macular atrophy is higher in patients with RAP lesions than patients with other types of CNV [6-8]. Studies that follow up patients for a prolonged period of time suggest that most patients will eventually develop atrophy or fibrosis and the risk increases with time [6]. Kim et al. evaluated the difference in long-term outcomes of patients with type 3 CNV regarding the development of geographic atrophy or fibrotic scars [9]. The authors show that disease stage and number of injections play a significant role in the emergence of macular atrophy; however, they do not include patients with stage 1 RAP lesions [9]. Baek et al. and Maruyama-Inoue et al. reported that almost 60% of their patients suffering from RAP developed eventually atrophy in a follow-up period of three and four years, respectively [6,10]. Both studies do not report any differences in the occurrence of macular atrophy between different stages of the disease. Patients in this study show no sign of macular atrophy after 24 months of follow-up period, indicating that early detection and fewer injections may lead to better anatomical outcomes.

Studies with a long-term follow-up period of patients with RAP suggest that visual acuity shows improvement or stabilisation during the first year and declines rapidly after that [6,7,11,12]. The CATT study suggests that patients with type 3 CNV experience a more significant decline in visual acuity compared to those with other types of CNV. As the study mentions, this decline cannot be solely attributed to a higher incidence of geographic atrophy [7]. However, these studies do not differentiate outcomes on visual acuity based on different stages of the disease. Kim et al. in two studies with only a 12-month follow-up period suggest that more advanced RAP stages produce worse visual outcomes, but the studies do not include patients with stage 1 RAP lesions [5,13]. Park and Roh findings are in accordance with the previous results but also include patients with stage 1 RAP, who produce better visual outcomes than more advanced stages [14]. However, they only follow up patients for 12 months, and as reported above, second and third year of follow-up are very crucial for the prediction of the final visual acuity (VA). Huang et al. in a three-year follow-up of patients with RAP lesions report worse visual outcomes in patients with stages 2 and 3 versus patients in stage 1 [15]. However, BCVA in patients with stage 1 RAP in the study declines to lower levels than baseline VA. In our study, all patients manage to maintain the same or even better visual acuity after a minimum of a 24-month follow-up period.

A recent review article authored by Mathis et al. presents various treatment approaches based on the disease stage [16]. The authors suggest that individuals with stage 1 RAP lesions should undergo a pro re nata regimen, as this approach leads to fewer recurrences and necessitates fewer injections to achieve improved outcomes [16]. Our findings align with this research, as patients in stage 1 RAP require only a limited number of injections and experience fewer recurrences when solely treated with anti-VEGF injections. Typically, maintaining satisfactory visual outcomes only requires vigilant monitoring. We propose that alternative treatment protocols like treat and extend or fixed dosing are not recommended for these patients due to their exceptional response during the initial loading dose.

The main limitations of this study are its retrospective design and small sample size. While our findings are consistent with previously reported data on the prognosis of early RAP lesions, larger studies comparing recurrence rates across all three stages are needed to better define differences in disease progression. We believe that treatment regimens should be tailored to the individual patient, taking these distinctions into account.

## Conclusions

The prognosis of patients with retinal angiomatous proliferation differs between different stages of the disease. By identifying the stage 1 lesion sooner, the study suggests that there could be reduced recurrences, less macular atrophy, and improved visual outcomes. Additionally, this early detection may have the potential to influence the treatment approach to these patients.
